# Quality of Life in a Swedish Cohort of Patients with Mycosis Fungoides

**DOI:** 10.2340/actadv.v106.adv-2026-0364

**Published:** 2026-04-22

**Authors:** Daria Hub, Kristina Drott, Emma Belfrage, Andreas Sonesson

**Affiliations:** 1 Division of Dermatology and Venereology, Department of Clinical Sciences, Lund University, Skåne University Hospital, Lund, Sweden; 2 Department of Haematology and Transfusion Medicine, Skåne University Hospital, Lund, Sweden

**Keywords:** CCL17, TARC, Cutaneous T-Cell Lymphoma, Health Related Quality of Life, Dermatology life quality index, Mycosis Fungoides, Itch, Skin

## Abstract

Mycosis fungoides (MF) is a disease that evolves slowly over years with few options for curative treatment. Improving health-related quality of life (HRQoL) is therefore an important treatment goal. This study aims to contribute to a better understanding of HRQoL, using a dermatology-adapted HRQoL questionnaire, Dermatology Life Quality Index (DLQI), in patients with MF. Baseline data from a cohort study (BIO-MUSE) of patients with MF were used to investigate which aspect of DLQI is the most affected and how DLQI relates to different clinical and physiological parameters. Data from a period of 12 months were used to study how DLQI changes over time. The symptom domain, for example itching, was the aspect of the DLQI that had the greatest impact on HRQoL. Thymus and activation-regulated chemokine/ chemokine (C-C) ligand 17 were analysed in blood and were shown to have a positive correlation with DLQI (*r*=0.482, *p*=0.043). It was further shown that increased itching at baseline was a strong predictor of a worse DLQI score in the domain of symptoms (*r*=0.677, *p*=0.002). Future research in a larger cohort is needed to investigate the effect of itch in HRQoL in patients with MF.

ClinicalTrials.gov: NCT04904146.

**Significance**

Mycosis fungoides (MF) is a cutaneous T-cell lymphoma with an often indolent course and few options for curative treatment. Hence, patients with MF often live with the diagnosis for many years. Therefore, the goal of treatment is to ensure stability of patients with MF. Our study showed that the disease’s symptoms, such as pruritus, are the factors that affect patients’ quality of life the most. Moreover, patients with higher levels of a signalling protein called thymus and activation-regulated chemokine/ chemokine (C-C) ligand 17 in the blood are in this study generally likely to have lower quality of life.

Cutaneous T-cell lymphomas (CTCLs) are a group of primary cutaneous lymphomas that are caused by a proliferation of malignant T-cells in the skin (1). CTCLs are a heterogeneous group of malignancies including the classic subtypes mycosis fungoides (MF) and Sézary syndrome (SS) (2, 3). MF accounts for approximately 60% of all CTCL cases, while SS accounts for around 5% (1). SS is an aggressive leukemic variant of CTCL, sharing the same TNMB classification, and often treatments, with advanced stages of MF (4).

The diagnosis of CTCL is determined by histopathology and molecular analysis of a skin biopsy in conjunction with the clinical presentation (2). Since the clinical picture of MF and SS can resemble nonmalignant inflammatory dermatoses and the histopathological features of MF can be minimal during the early stages of the disease, it can often be challenging to make the correct diagnosis (2). Many patients with MF often have a diagnostic delay of several years (5, 6). The TNMB classifications of MF and SS are defined by the International Society for Cutaneous Lymphomas (7). Most patients with MF present with early stage of disease (stage IA–IIA) (8). MF has a peak age between 50 and 74 years, with an incidence that is higher in men than women (4, 9). The annual CTCL incidence has been estimated to be approximately 0.7 per 100,000 (9). The first symptoms of the disease can be scaling patches, but it can present as thicker plaque, tumour or erythroderma (1).

Health-related quality of life (HRQoL) can be described as a person’s perceived wellbeing in physical, social and mental aspects of health (10). For example, symptoms such as sleep disturbance, the disease affecting their choice of clothes or causing frustration, anger and health distress, such as worrying about the prognosis, all affect HRQoL in patients with CTCL (11). Pruritus has been reported to be the most frequently reported symptom of MF and also the symptom that affects the patients’ HRQoL the most (12). In qualitative studies of HRQoL in patients with MF, it is often reported that the patients feel that if they had received an earlier diagnosis, it could have reduced the progression of their disease (12). Patients have also reported frustration from having a rare disease that the general population has little knowledge about, and that they often have to encounter the misperception of their disease being contagious since it contains the name fungus (12).

CC chemokine receptor 4 (CCR4) is expressed mainly on certain Th2 cell subsets and on malignant T-cells, such as adult T-cell leukaemia/lymphoma and CTCL (13). Thymus and activation-regulated chemokine/chemokine (C-C) ligand 17 (TARC/CCL17) is one of the ligands of CCR4 and acts as a chemoattractant stimulating migration of cells. TARC/CCL17 was therefore chosen to be part of the physiological parameters in this study (13). In patients with atopic dermatitis, a positive correlation has been shown between changes in TARC/CCL17 levels and changes in pruritus score (14). In a study from 2001, the average value for TARC/CCL17 in serum of patients with atopic dermatitis was significantly higher than for healthy subjects and patients with psoriasis (15).

Improving HRQoL is one of the treatment goals for patients with MF, and therefore, it is important to further increase knowledge about quality of life in this patient group. The Dermatology Life Quality Index (DLQI) is a widely used tool to assess the impact of a dermatological disease on HRQoL and was first published in 1994 (11, 12, 16).

In a systematic review from 2021 on quality of life in patients with MF, several studies had used DLQI, but none of the studies had analysed the questionnaire’s different components, and which aspects of quality of life were the most affected domain (12, 17–20). This study aimed to further investigate HRQoL in patients with MF and analyse in which aspects their quality of life is impaired and how DLQI relates to different clinical and physiological parameters.

## MATERIALS AND METHODS

### Study design

The BIO-MUSE study, ClinicalTrials.gov NCT04904146, is a prospective translational study. This part of the study consists of data from 18 patients with MF (21). The patients were enrolled from 2021 to 2023 and were planned to be part of the BIO-MUSE study for 3 years. The patients received treatment according to clinical routine. Sampling of each patient was performed every 3 months. Three patients died during the study, and all causes were related to MF. Two patients decided to leave the study. Inclusion and exclusion criteria, and other aspects of the study are further described in the study protocol (21). Informed written consent was obtained from all participants according to ICH-GCP guidelines and the Declaration of Helsinki. The study was approved by the Swedish Ethical Review Authority, DNR 2019–05130.

### Clinical parameters

*Dermatology Life Quality Index (DLQI).* This HRQoL questionnaire is specifically developed to assess the impact of dermatological diseases (16, 22). The questions concern symptoms (question 1), feelings (question 2), daily activities (questions 3 and 4), leisure (questions 5 and 6), work/school (question 7), relationships (questions 8 and 9) and treatment (question 10) (22). Each question allows the patient to answer whether their disease affects them “not at all=0 points”, “a little=1 point”, “somewhat=2 points” or “very much=3 points”, and together, this produces a score from 0 (no impairment on quality of life) to 30 (maximum impairment) (16, 22). The patient can also answer “not relevant”, which is scored as zero points (22). When interpreting the overall DLQI score, the following guidelines can be used: “21–30 points=extremely large impact on patient’s life”, “11–20=very large impact”, “6–10=moderate impact”, “2–5=small impact” and “0–1=no impact” (23). The questionnaire assesses the impact on the patient’s quality of life in the previous week (16). A study from 2015 recommended that the minimal clinically important difference (MCID) for DLQI should be 4 (24).

*Modified Severity Weighted Assessment Tool (mSWAT).* The SWAT score was originally developed for clinicians to have a quantitative tool to assess the severity of MF (25). It was designed to be used in both clinical trials and assessment of individual patients and is obtained by multiplying the body surface area of each skin lesion by a weighting factor (1 for patch, 2 for plaque and 3 for tumour) (25). The mSWAT is calculated using the same method but uses altered weighting factors (1 for patch, 2 for plaque and 4 for tumours) (26).

*Pruritus Numeric Rating Scale (Pruritus NRS).* The peak pruritus NRS is designed to measure the patient’s worst itch over the last 24 h (27). In this study, the patients rated their worst pruritus on a scale from zero to ten where “0=no pruritus” and “10=worst pruritus imaginable”.

*Sleep Numeric Rating Scale (Sleep NRS).* The sleep NRS measured sleep from the patient’s previous week and ranged from 0 to 10 where “0=good sleep” and “10=very disrupted sleep”. In a study on patients with atopic dermatitis, it was reported that the sleep disturbance NRS is easy for patients to understand (28).

### Physiological parameters

*Transepidermal water loss (TEWL*). TEWL is a measurement of water loss that arises when water diffuses over the stratum corneum and is measured with the unit g/m^2^h (29). Skin diseases where the skin barrier is damaged are associated with a higher TEWL than that for healthy skin (29). The TEWL value is dependent on the skin area of the body; breast skin, for example, has a lower TEWL than skin from the axilla (30). In this study, TEWL was measured according to guidelines, using a closed-chamber TEWL meter, VapoMeter 300 (31). The upper arm was chosen for TEWL measurements when possible.

*Chemokine ligand-17 (CCL17).* Analyses of TARC/CCL17 in blood were performed at the Department of Clinical Immunology at Skåne University Hospital.

### Statistical analysis

The statistical analyses were performed in SPSS. The Mann–Whitney test was used to compare the mean rank between men and women. Spearman rank correlation was chosen for nonparametric measure. A correlation coefficient of <0.40 was considered weak, 0.40–0.59 moderate, 0.60–0.80 strong and 0.80–1.0 very strong. When comparing the median value at baseline and after 12 months, the Wilcoxon signed-rank test was used. Results with *p*-values below 0.05 were considered statistically significant.

## RESULTS

### Study population

Eighteen patients with MF were included in the study. The patients had TNMB stage ranging from stage IA to IIIB, but the majority (78%) had early stages of disease (stage IA–IIA). Among male patients, 82% had early stage of disease and in female patients 7 1% had early stage of disease. Although the study intended to include both patients with MF and SS, no patients with SS were eligible for inclusion. The included patients were between 31 and 85 years old with a median age of 71.5 years. This study consisted of 11 (61%) men and 7 (39%) women.

### DLQI scores of study population

**Fig. 1.**
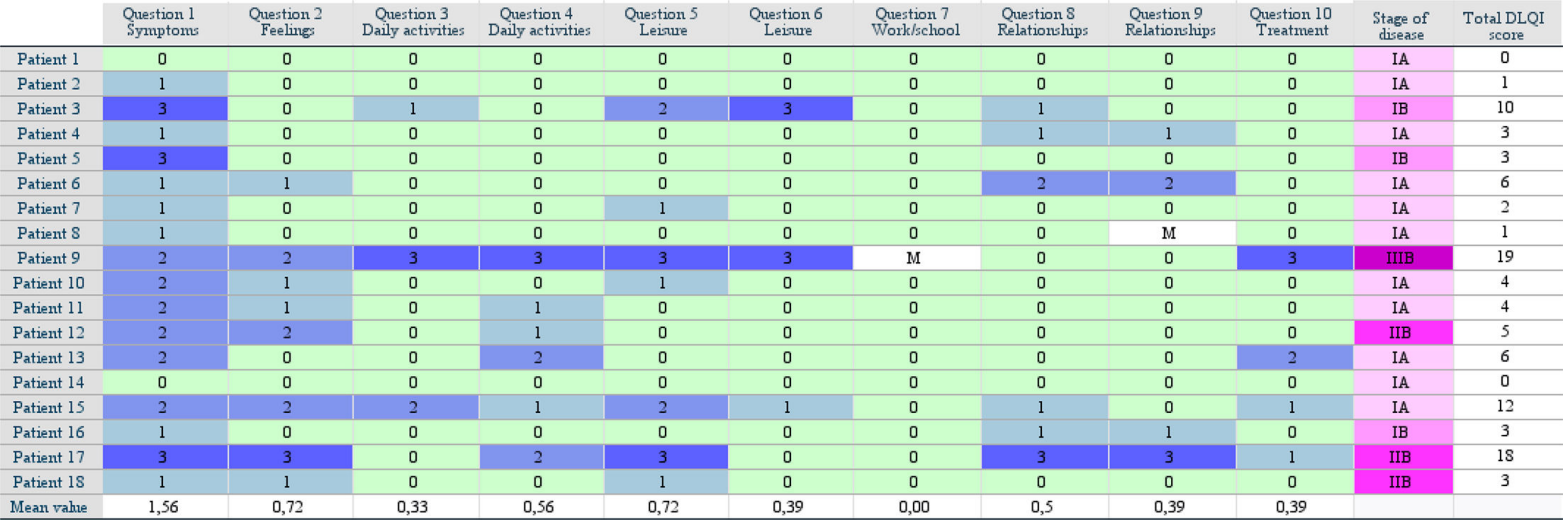
Heatmap of DLQI scores for each patient and question. DLQI: Dermatology Life Quality Index; M: missing value.

**Fig. 2.**
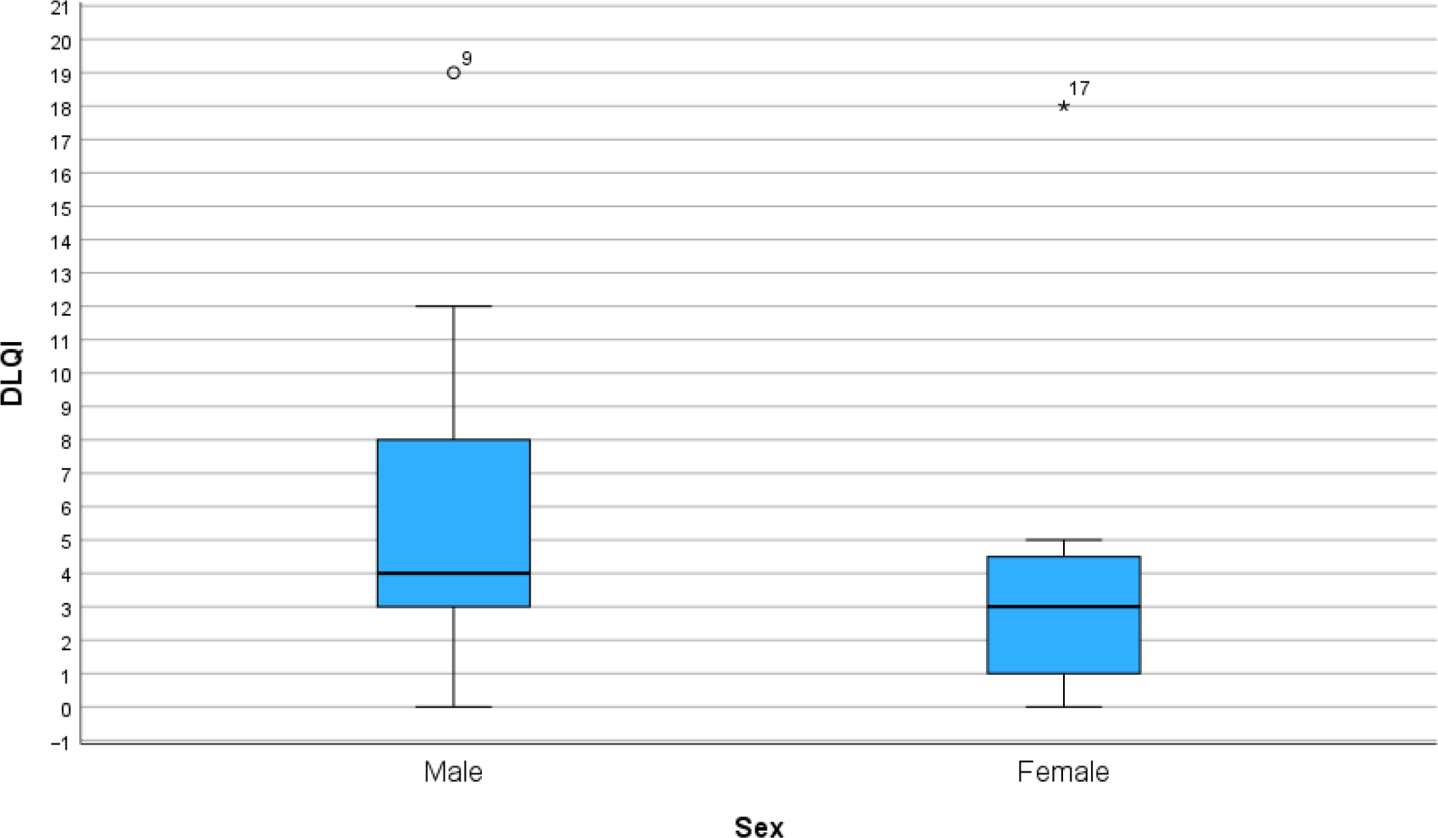
DLQI for men and women. The difference in median value between men and women was not statistically significant (*p*=0.382) (Mann–Whitney test). DLQI, Dermatology Life Quality Index; °/* display outliers.

Answers from DLQI questionnaires from the 18 patients at baseline were collected and are displayed in the form of a heatmap (Fig. 1). The total DLQI score for each patient ranged from 0 to 19 points. The cohort had a median DLQI of 3.5 points and a mean value of 5.6 points. Looking at each question individually, question 1 (regarding symptoms) had a mean value of 1.56 points in the study cohort, which is greater than the mean value of the other questions. Questions 2 (regarding feelings) and 5 (regarding leisure) had a mean value of 0.72 points. The median value for DLQI was 4 for men and 3 for women in the studied population (Fig. 2). The difference between men and women was not statistically significant.

### DLQI related to clinical and physiological parameters

When studying how DLQI related to different clinical and physiological parameters, TARC/CCL17 at baseline turned out to be a moderate predictor of DLQI (*r*=0.482, *p*=0.043) (Table I, Fig. S1). Moreover, the pruritus NRS at baseline was a strong predictor of DLQI question 1, the domain of symptoms, (*r*=0.677, *p*=0.002) (Table II, Fig. S2). No significant correlations were found between DLQI and other parameters (Table I).

**Table I.** Correlation between DLQI and different clinical and physiological parameters

			DLQI
Spearman’s rho	DLQI	Correlation coefficient	1,000
		Sig. (2-tailed)	.
		N	18
	TARC/CCL17	Correlation coefficient	0,482^*^
		Sig. (2-tailed)	0,043
		N	18
	Pruritus according to NRS	Correlation coefficient	0,466
		Sig. (2-tailed)	0,051
		N	18
	Sleep disturbance according to NRS	Correlation coefficient	0,461
		Sig. (2-tailed)	0,054
		N	18
	Age	Correlation coefficient	0,334
		Sig. (2-tailed)	0,176
		N	18
	TEWL	Correlation coefficient	0,031
		Sig. (2-tailed)	0,904
		N	18
	mSWAT score	Correlation coefficient	0,234
		Sig. (2-tailed)	0,350
		N	18
	Stage of disease	Correlation coefficient	0,458
		Sig. (2-tailed)	0,056
		N	18

* Correlation is significant at the 0.05 level (2-tailed).

DLQI:dermatology life quality index; mSWAT:modified severity weighted assessment tool; N:number; NRS:numeric rating scale; TARC/CCL17:thymus and activation regulated chemokine/CC chemokine-ligand-17; TEWL:transepidermal water loss.

**Table II.** Correlation between DLQI question 1, in the domain of symptoms, and different clinical and physiological parameters

			DLQI question 1
Spearman’s rho	DLQI question 1	Correlation coefficient	1,000
		Sig. (2-tailed)	.
		N	18
	TARC/CCL17	Correlation coefficient	0,366
		Sig. (2-tailed)	0,135
		N	18
	Pruritus according to NRS	Correlation coefficient	0,677^**^
		Sig. (2-tailed)	0,002
		N	18
	Sleep disturbance according to NRS	Correlation coefficient	0,331
		Sig. (2-tailed)	0,180
		N	18
	Age	Correlation coefficient	0,229
		Sig. (2-tailed)	0,360
		N	18
	TEWL	Correlation coefficient	−0,105
		Sig. (2-tailed)	0,680
		N	18
	mSWAT score	Correlation coefficient	0,438
		Sig. (2-tailed)	0,069
		N	18
	Stage of disease	Correlation coefficient	0,464
		Sig. (2-tailed)	0,053
		N	18

** Correlation is significant at the 0.01 level (2-tailed).

DLQI question 1:dermatology life quality index question 1; mSWAT:modified severity-weighted assessment tool; N:number; NRS:numeric rating scale; TARC/CCL17:thymus and activation regulated chemokine/CC chemokine-ligand-17; TEWL:transepidermal water loss.

### DLQI changes in the study cohort over the period of one year

**Fig. 3.**
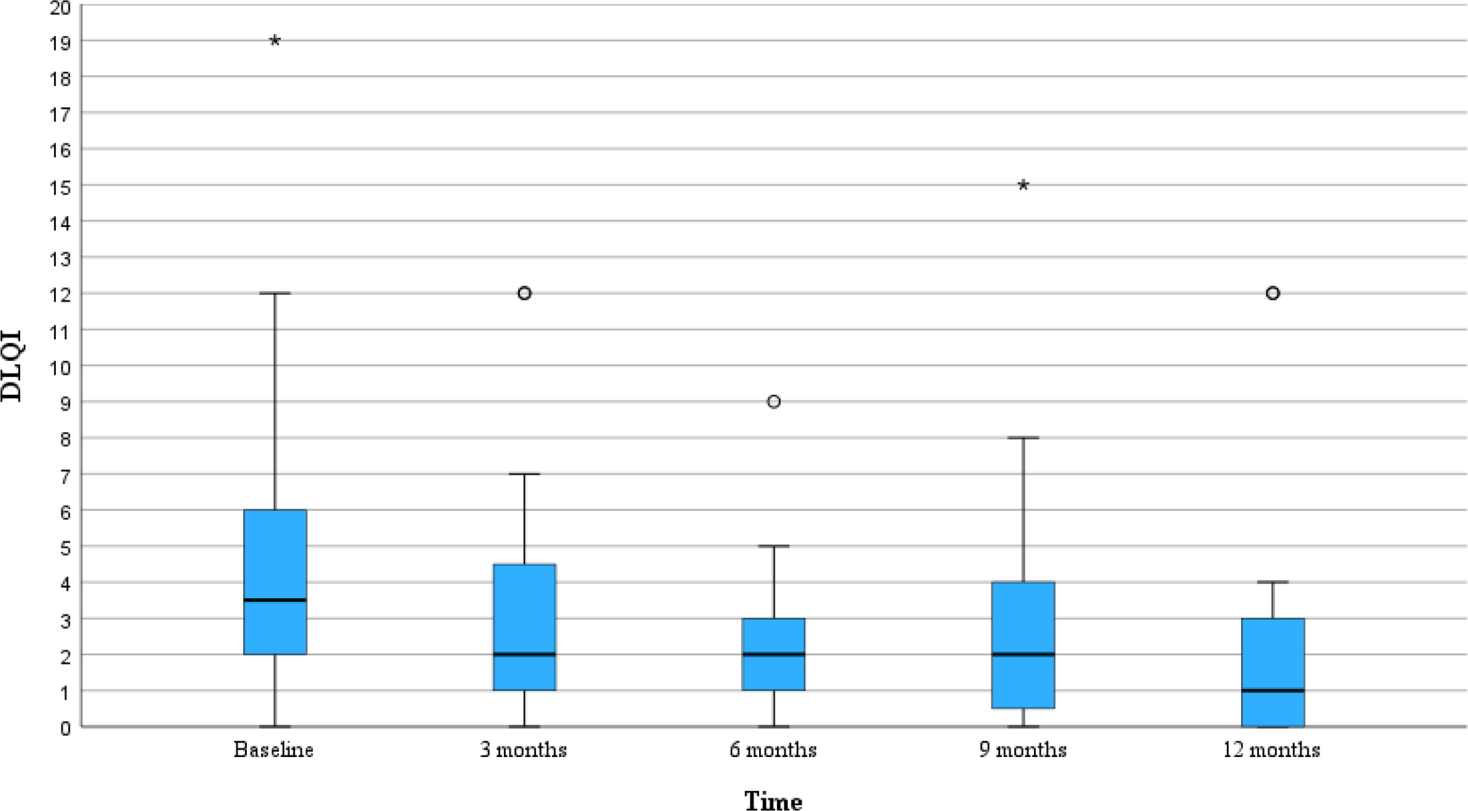
DLQI displayed over a period of 12 months. The DLQI changes over 12 months in the cohort. There was no significant improvement of DLQI between baseline and 12 months follow-up (*p*=0.481) (Wilcoxon signed-rank test). DLQI, Dermatology Life Quality Index; °/* display outliers.

To evaluate whether the DLQI in this cohort changed over a period of 12 months, DLQI was recorded at baseline and every 3 months, for 12 months. The results showed that the median value varied during the investigated period (Fig. 3). However, the difference in DLQI score at baseline and at 12 months in this cohort was not statistically significant.

## DISCUSSION

This study showed that the disease’s symptoms, such as pruritus, are the factors that most affect quality of life in patients with MF. Interestingly, there was a positive correlation between DLQI question 1 (regarding symptoms) and the pruritus’ NRS. In the cohort, the median DLQI score varied during the investigated period of 12 months, although no significant improvement could be found during that time. In the studied cohort, a median DLQI score of 3.5 points was found at baseline, which is consistent with results of patients with MF reported in other studies. In a study from 2019, Martin-Carrasco et al. (32) reported a mean DLQI score of 3.87 points among 53 patients with CTCL. In this cohort, all patients had MF, with most of the cases being early stage. In another study, the mean DLQI score was 9.93 points, assessed in 30 MF patients with early-stage disease; this was higher compared to our cohort (33). In a systematic review, the mean and median DLQI ranged between 3 and 20.4 points for studies done on MF and SS (12). Consistent with the results of the present study, Martin-Carrasco et al. found that the question regarding symptoms was the most affected aspect of DLQI (32).

Our results from the DLQI question about symptoms (question 1) can be compared to other studies that have used dermatology-specific questionnaires for HRQoL in patients with CTCL. The systematic review by Ottevanger et al. (12) included studies that used the dermatology-specific questionnaire Skindex-29. Skindex-29 is divided into the domains of “symptoms”, “emotions” and “functioning” (12). Seven of the included studies with Skindex-29 reported the results by showing the different domains of the questionnaire (12). Three of the seven studies reported that “symptoms” was the most affected domain and one study reported that “emotions” and “symptoms” were almost equally affected (12). Both Martin-Carrasco et al. and Ottevanger et al. performed studies that are aligned with the results of the present study (12, 32).

However, when comparing median DLQI between men and women, the results of our study do not align with other studies. In our cohort, there were no statistically significant differences in DLQI between men and women. Chalaka et al. (34), on the contrary, found that HRQoL was worse in women compared to men, even when adjusting for stage of disease. Stefanie Porkert et al. (35), on the other hand, gathered results that showed that HRQoL was not related to gender. These findings show that there is a need to further study how HRQoL relates to gender. In a systematic review, Ottevanger et al. (12) showed that HRQoL is impaired to a higher degree in patients with advanced stage of disease compared to early stages of disease. Other studies found that HRQoL is worse in advanced stages of disease compared to early stages of disease (12, 36). Moreover, the study of Berna Solak et al. (37) found a positive correlation between pruritus and total DLQI score in a cohort of 81 patients with early-stage MF. However, in our study, the number of individuals included was small and the results presented here could not confirm an association between pruritus and total DLQI score.

Interestingly, a positive correlation was shown between DLQI and TARC/CCL17. An explanation for this may be that TARC/CCL17 is associated with an increased disease activity and type 2 inflammation, which likely contributes to pruritus, which is part of the symptom domain of the DLQI, in a similar way as in atopic dermatitis (14, 15). Future studies and more knowledge are needed to understand in more detail the role of TARC/CCL17 in relation to HRQoL of MF patients.

We conclude that the disease’s symptoms, such as pruritus, are the factors that affect patients’ quality of life the most. It would be interesting to further investigate the relationship between pruritus and DLQI in patients with MF through a qualitative study, to clarify the relationship in more detail. Moreover, these results indicate that higher levels of TARC/CCL17 in the blood could be associated with more pruritus and a lower quality of life. However, more research is needed in a larger cohort to investigate the causes and motives behind these associations in terms of HRQoL in patients with MF.
