# Supplementary material for: Quality of Life in a Swedish Cohort of Patients with Mycosis Fungoides
Source: Acta Derm Venereol. 2026 Apr 22;106:0364. doi: 10.2340/actadv.v106.adv-2026-0364 (PMC13103715; doi:10.2340/actadv.v106.adv-2026-0364)
Supplement: Supplementary file 1. [file ActaDv-106-0364-s0001.pdf]

SUPPLEMENTAL FIGURE LEGENDS

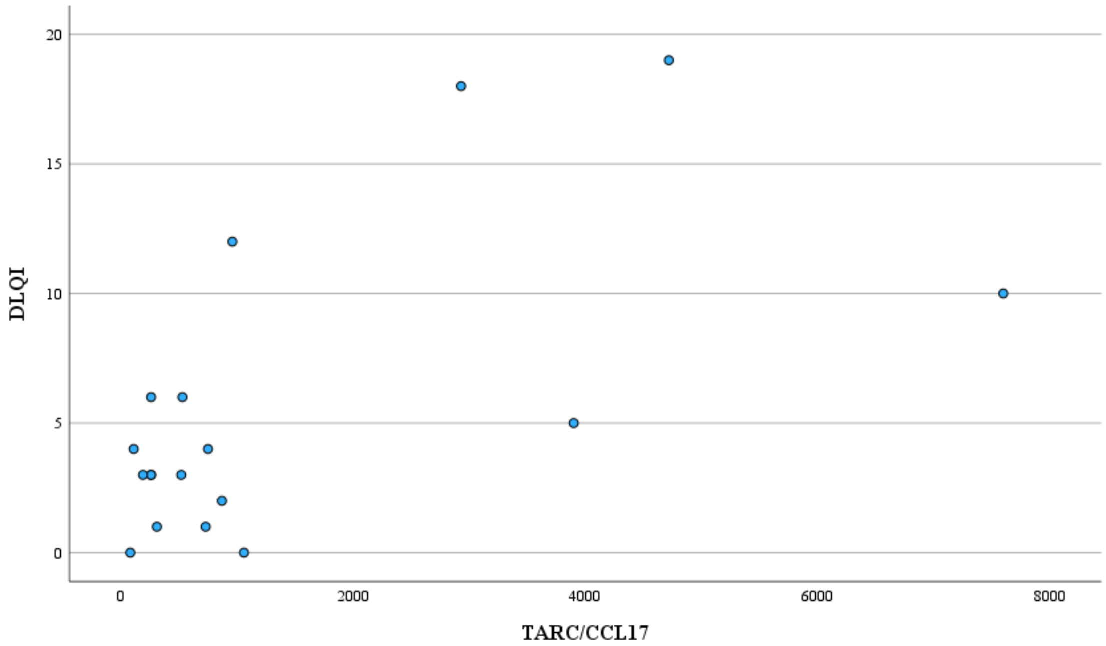

Fig. S1. Scatter plot showing the relationship between TARC/CCL17 and DLQI. TARC/CCL17 at baseline was a moderate predictor of DLQI. *DLQI, dermatology life quality index; TARC/CCL17, thymus and activation regulated chemokine/ CC chemokine-ligand-17.*

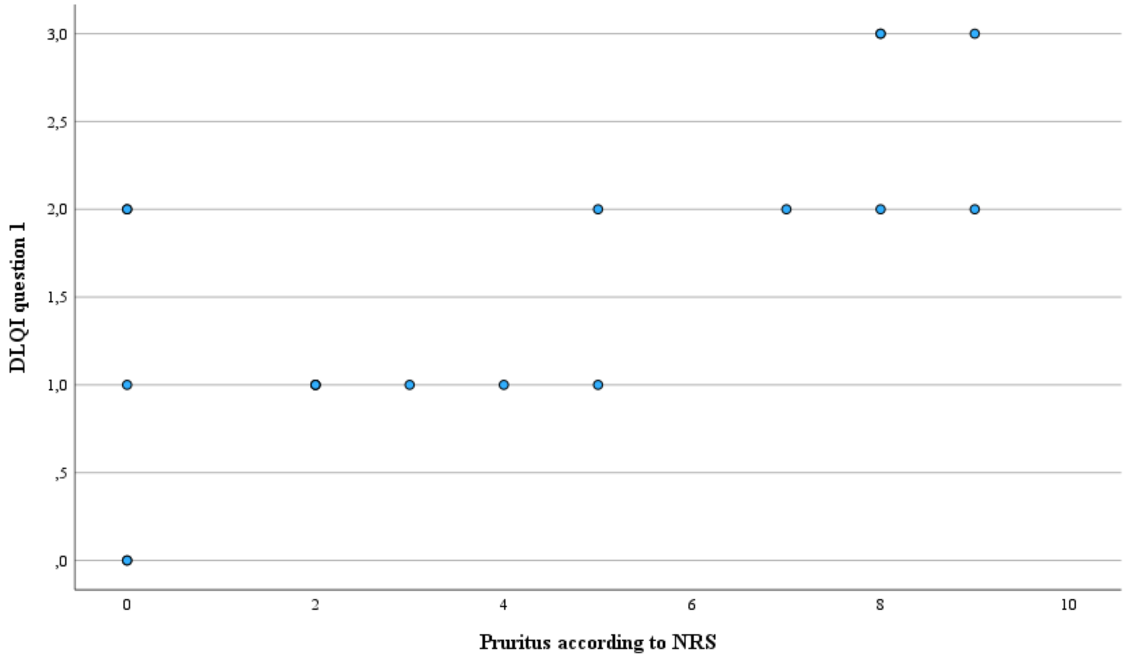

Fig. S2. Scatter plot showing the relationship between DLQI question 1 and pruritus according to NRS. The pruritus NRS at baseline was a strong predictor of the DLQI domain of symptoms (question 1). *DLQI, dermatology life quality index; NRS, numeric rating scale.*
